# Factors affecting marriage decisions among couples with sickle cell or beta-thalassemia traits

**DOI:** 10.1016/j.jtumed.2025.08.003

**Published:** 2025-09-08

**Authors:** Mohammed Badedi, Abdulrahman Muidh, Mannaa Alsayed, Hussain Darraj, Ali Shubaili, Abdulaziz Darraj, Yahya Hobani, Musa Ahmadini, Ibrahim Hakami, Nabeel Gharawi, Mohammed Hakami, Khalid Shrwani, Nhari Malhan, Asma Mokali, Ahmed Abdaly

**Affiliations:** a Public Health, Ministry of Health, KSA; b Jazan Health Cluster, KSA

**Keywords:** At-risk marriages, Premarital screening, Sickle cell disease, Thalassemia, الفحص الطبي قبل الزواج, الزيجات عالية الخطورة, أنيميا البحر المتوسط, فقر الدم المنجلي

## Abstract

**Objective:**

This study assessed the prevalence of marriage among genetically incompatible couples at risk of having children with either sickle cell disease (SCD) or beta-thalassemia (β-thalassemia) in Jazan province, KSA, and the factors influencing these couples’ decisions to marry.

**Methods:**

An analytical cross-sectional research design was used. From the National Health Premarital Screening System database, couples whose premarital screening tests indicated genetic incompatibility and a risk of having children with either SCD or β-thalassemia were randomly selected, with a sample size of 386 couples.

**Results:**

The prevalence of risky marriages that might result in children with SCD or β-thalassemia was 60.4 %. Marriage was more favored among participants who already were married, divorced, or widowed, or who had children during the screening period, than among unmarried participants (P = 0.006). Couples with good knowledge and awareness of the risks of SCD to children's lives decided not to marry (P = 0.001). Furthermore, couples who attended all relevant counseling sessions were more likely to decide not to marry than those who attended only one counseling session (P = 0.001). Despite the positive effects of these counseling sessions, many social, cultural, and religious factors, most prominently religion (70.8 %), influenced these couples' decisions to marry and accept the possibility of having a child with SCD or β-thalassemia.

**Conclusion:**

Given the high prevalence of SCD and at-risk marriages in the Jazan region, in vitro fertilization and pre-implantation genetic testing services are recommended.

## الملخص

**أهداف البحث:**

تهدف هذه الدراسة إلى تقييم مدى انتشار الزواج بين الأزواج غير المتوافقين وراثيًا، والمُعرّضين لخطر إنجاب أطفال مصابين بفقر الدم المنجلي أو أنيميا البحر المتوسط في منطقة جازان بالمملكة العربية السعودية، بالإضافة إلى تقييم العوامل المؤثرة في قرار هؤلاء الأزواج بالإقدام على الزواج.

**طرق البحث:**

تم اعتماد تصميم دراسة مقطعية تحليلية. واستُخلصت العينة من قاعدة بيانات برنامج الفحص الطبي قبل الزواج الوطني، حيث جرى اختيار 386 زوجًا بشكل عشوائي من بين الأزواج الذين أظهرت نتائج فحوصاتهم وجود عدم توافق وراثي واحتمالية إنجاب أطفال مصابين بفقر الدم المنجلي أو أنيميا البحر المتوسط.

**النتائج:**

بلغ معدل انتشار الزيجات عالية الخطورة التي قد تؤدي إلى إنجاب أطفال مصابين بفقر الدم المنجلي أو أنيميا البحر المتوسط نحو 60.4%. وقد كان الإقدام على الزواج أكثر شيوعًا بين المشاركين الذين كانوا متزوجين أو مطلقين أو أرامل، أو ممن لديهم أبناء خلال فترة الفحص، مقارنةً بغير المتزوجين .(P = 0.006)كما أظهرت النتائج أن الأزواج الذين يمتلكون معرفة ووعيًا جيدًا بمخاطر فقر الدم المنجلي على حياة الأطفال كانوا أكثر ميلًا لاتخاذ قرار بعدم الزواج .(P = 0.001) إضافة إلى ذلك، فإن الأزواج الذين حضروا جميع جلسات المشورة الوراثية ذات الصلة كانوا أكثر احتمالًا لاتخاذ قرار بعدم الزواج مقارنةً بمن حضروا جلسة واحدة فقط .(P = 0.001) وعلى الرغم من التأثير الإيجابي لتلك الجلسات، فقد كان للعديد من العوامل الاجتماعية والثقافية والدينية ، وعلى رأسها العامل الديني بنسبة 70.8% ، دورٌ مؤثرٌ في قرار الأزواج بالمضي قدمًا في الزواج وقبول احتمالية إنجاب طفل مصاب.

**الاستنتاجات:**

نظرًا للانتشار المرتفع لحالات فقر الدم المنجلي وزيجات الخطورة الوراثية في منطقة جازان، توصي الدراسة بتوفير خدمات الإخصاب خارج الجسم وفحص الأجنة قبل التخصيب كخيارات وقائية للحد من هذه الأمراض الوراثية.

## Introduction

Sickle cell disease (SCD) and beta-thalassemia (β-thalassemia) are inherited hemoglobin disorders that cause substantial morbidity and mortality and pose a financial burden on society.^1^ Among the approximately 5.2 % of the global population with these disorders, 4.4 % are of Eastern Mediterranean origin.^2^ In the Kingdom of Saudi Arabia (KSA), approximately 4.2 % of the population carries the sickle cell trait, and 0.26 % has SCD.^3^ Consequently, SCD and β-thalassemia are targeted in premarital screening before marriage license issuance.^4^ Premarital screening is compulsory in KSA to identify couples with genetic diseases, particularly hemoglobinopathies, such as SCD and β-thalassemia.^4^ Genetically incompatible (i.e., at-risk) couples can then be advised regarding their possibility of bearing affected children, to decrease marriage between such couples and the incidence of both diseases in the population. At-risk couples remain free to marry and have children regardless of their test results, but they are counseled regarding the advisability of in vitro fertilization and pre-implantation genetic testing.^4^ Premarital screening services are provided by more than 300 centers across KSA, and include laboratory testing and medical consultation sessions for common genetic blood disorders, such as SCD and β-thalassemia.^4^

Many factors affect marriage decisions among at-risk couples, including religious beliefs, cultural norms, traditions, literacy, education, government policy, and the attitudes of the couples themselves. However, research is lacking regarding the prevalence of at-risk marriages and the factors influencing marriage decisions among couples with sickle cell or β-thalassemia traits in the southwestern province of Jazan in KSA, which has a high prevalence of inherited hemoglobinopathies.^3^ This study was aimed at measuring the prevalence of married at-risk couples in Jazan province, and determining the factors that influenced their decisions to marry despite premarital screening results indicating their genetic incompatibility and their SCD or β-thalassemia carrier status.

## Materials and Methods

### Research design

#### Study design and sampling technique

This analytical cross-sectional study included 386 Saudi couples who had undergone premarital screening tests in Jazan province between 2021 and 2023 and were found to be genetically incompatible regarding SCD or β-thalassemia. Jazan is a small city in southwestern KSA with a relatively homogeneous population with similar ethnic and socioeconomic characteristics. We first identified all such couples from the National Health Premarital Screening System database in the described period as a sampling frame. Subsequently, we selected participants through a simple randomization technique.

#### Sample size

The sample size was estimated in Epi Info software with a confidence interval of 95 %, power of 80 %, and expected prevalence 50 %. On the basis of these parameters, the estimated minimum sample size required was determined to be 384 couples. However, we added 10 % for non-response, thus resulting in a target sample size of 422 participating couples. A total of 286 couples participated in the study (response rate 91.5 %).

### Data collection

Each couple was interviewed by telephone for an average time of 10 min. Their oral informed consent was obtained before each interview. A questionnaire was designed to collect the following information: confirmation of decisions to marry or not marry; premarital screening test results; sociodemographic and medical history data, including age, district, education level, marital status, number of children at the time of premarital screening, occupation, average monthly income, presence of comorbidities (diabetes, cancer, chronic heart disease, etc.), familial relationship to the partner; family history of SCD or β-thalassemia; knowledge of premarital screening and its purpose, and source of this knowledge; effects of counseling services on decisions to marry; and reasons for, and factors that influenced, the decision to marry despite genetically incompatible premarital screening test results for SCD or β-thalassemia.

### Data analysis

Data entry and analysis were performed by the principal investigator in Statistical Package for the Social Sciences software version 24. To ensure the privacy of the participants, the data were coded with anonymous identification numbers. After data screening and assumption checks, no data were missing, and the normality of the distribution of the variables was determined with the Shapiro–Wilk test. Categorical variables are presented as counts and percentages, whereas continuous variables are presented as means ± standard deviations. The chi-square test was used to detect significant associations between categorical variables, on the basis of verification of the assumptions of expected cell counts >5 in at least 80 % of cells and expected counts <5 in no more than 20 % of cells. If these assumptions were violated, Fisher's exact test was used as an alternative. Post-hoc pairwise comparisons were used to assess differences between categorical variables that showed statistically significant associations after Bonferroni correction. Independent samples t-test was used to compare differences in mean values between groups after verification of the normality assumption. (Homogeneity of variance was verified with Levene's test, and Welch's t-test was used if violation was found.) The Mann–Whitney U test was not used, because no normality violations were found. The significance threshold was set at p < 0.05.

## Results

Of the 386 at-risk couples recruited for this study, 233 (60.4 %) were married, and 153 (39.6 %) were not married. The premarital screening test results for all 772 male and female participants were as follows: 639 (82.8 %) had sickle cell traits (i.e., were SCD carriers), 109 (14.1 %) had β-thalassemia traits (i.e., were β-thalassemia carriers), 21 (2.7 %) had SCD, and only 1 (0.4 %) had β-thalassemia. Genetically incompatible results showed no statistically significant association with the couples’ decisions to marry (P-value = 0.057).

### Sociodemographic characteristics

Table 1 shows the sociodemographic characteristics of the study population, stratified according to marriage decision. The mean age of the men was 31.1 ± 8.5 years (range: 18–71), and that of the women was 26 ± 7.2 years (range: 14–65). Married and unmarried participants showed no statistically significant difference in mean age. Most participants (64.2 %) had completed education as high as secondary education. Although the unmarried participants had higher education levels (i.e., university degrees) than the married participants, education level was not significantly associated with decisions to marry. More participants who lived in the south and east sectors of Jazan were married than those who lived in other sectors of the province. The following participants showed a predisposition to marriage: those with high income (≥10,000 SR); those who were married, divorced, or widowed; those who had children (odds ratio [OR] = 2.2); and those who were retired. Approximately 56.5 % of the couples in the study population were unrelated; this group had a significantly lower probability of marrying than the first-degree blood-related couples. Among participants with a family history of SCD or β-thalassemia, 49 % decided not to marry.

**Table 1:** Sociodemographic characteristics and marriage decisions.

Variables	Overall = 386 n (%)	Did not marry = 153 n (%)	Did marry = 233 n (%)	*P*
**Age in years (mean ± SD)**				
Male age	31.1 ± 8.5	30.1 ± 7.3	31.7 ± 9.2	0.063
Female age	26.0 ± 7.2	25.2 ± 6.6	26.6 ± 7.5	0.072
Age difference between partners	5.9 ± 5.1	5.8 ± 4.7	5.9 ± 5.3	0.793
**Education level**				
Not educated	2 (0.5)	1 (0.7)	1 (0.4)	0.282
Primary/secondary	248 (64.3)	91 (59.5)	157 (67.4)	
University	136 (35.2)	61 (39.8)	75 (32.2)	
**Residence sector**				
North sector	61 (15.8)	23 (15.0)	38 (16.3)	0.282
South sector	98 (25.4)	35 (22.9)	63 (27.0)	
West sector	91 (23.6)	45 (29.4)	46 (19.8)	
East sector	103 (26.7)	39 (25.5)	64 (27.5)	
Center sector	33 (8.5)	11 (7.2)	22 (9.4)	
**Marital status at screening**				
Never married	302 (78.2)	133 (86.9)	169 (72.6)	0.006^a^
Married	79 (20.5)	20 (13.1)	59 (25.3)	
Divorced	4 (1.0)	0	4 (1.7)	
Widowed	1 (0.3)	0	1 (0.4)	
**Children at time of screening**				
No children	319 (82.6)	136 (88.9)	183 (78.5)	0.009^a^
Children	67 (17.4)	17 (11.1)	50 (21.5)	
**Occupation**				
Not working	25 (6.5)	9 (5.9)	16 (6.8)	0.555
Student	19 (4.9)	9 (5.9)	10 (4.3)	
Governmental/private	326 (84.5)	131 (85.6)	195 (83.7)	
Retired	16 (4.1)	4 (2.6)	12 (5.2)	
**Average monthly income**				
<5000 SR	105 (27.2)	37 (24.2)	68 (29.2)	0.006^a^
5000–10,000 SR	209 (54.1)	97 (63.4)	112 (48.1)	
>10,000 SR	72 (18.7)	19 (12.4)	53 (22.7)	
**Presence of comorbidities**				
No	360 (93.3)	142 (92.8)	218 (93.6)	0.773
Yes	26 (6.7)	11 (7.2)	15 (6.4)	
**Blood relationship (consanguinity)**				
None	218 (56.5)	91 (59.5)	127 (54.5)	0.475
Third degree (first cousin)	117 (30.3)	41 (26.8)	76 (32.6)	
Fourth degree (second cousin)	51 (13.2)	21 (13.7)	30 (12.9)	
**Family history of sickle cell anemia/β-thalassemia**				
No	198 (51.3)	69 (45.1)	129 (55.4)	0.142
Yes	168 (43.5)	75 (49.0)	93 (39.9)	
Do not know	20 (5.2)	9 (5.9)	11 (4.7)	

a Significant results (P value < 0.05).

### Knowledge of compulsory screening

Most (97.4 %) participants were aware that premarital screening is compulsory in KSA (Table 2). However, participants who did not understand the purpose of premarital screening were more likely to marry (OR = 2), as were those who did not know about hereditary diseases and their risk to children's lives (OR = 2.9). Most participants (69.2 %) had gained knowledge regarding premarital screening and its purpose from the provincial healthcare services (Table 2). Approximately 66.1 % of the participants suggested that at-risk couples should undergo premarital screening tests before engagement. Post-hoc comparisons indicated that the monthly income category of 5000–10,000 SR significantly differed from the >10,000 SR category. Participants with an income >10,000 SR had a higher likehood of marriage (see Table 3).

**Table 2:** Participants’ knowledge and awareness of sickle cell anemia in marriage decisions.

Variables	Overall = 386 n (%)	Did not marry = 153 n (%)	Did marry = 233 n (%)	*P*
**Awareness of compulsory screening**				
Unaware	10 (2.6)	7 (4.6)	3 (1.3)	0.047
Aware	376 (97.4)	146 (95.4)	230 (98.7)	
**Understanding the purpose of screening**				
Do not know/not sure/low knowledge	325 (84.2)	121 (79.1)	204 (87.6)	0.026
Good knowledge	61 (15.8)	32 (20.9)	29 (12.4)	
**Knowledge regarding the premarital screening tests**				
Lack of knowledge	322 (83.4)	122 (79.7)	200 (85.8)	0.115
Good knowledge	64 (16.6)	31 (20.3)	33 (14.2)	
**Awareness regarding disease prevalence in Jazan**				
Unaware	92 (23.8)	37 (24.2)	55 (23.6)	0.896
Aware	294 (76.2)	116 (75.8)	178 (76.4)	
**Knowledge regarding disease hereditary**				
Lack of knowledge	184 (47.7)	60 (39.2)	142 (60.9)	0.001^a^
Good knowledge	202 (52.3)	93 (60.8)	91 (39.1)	
**Awareness regarding the risk of diseases to children's lives**				
Unaware	21 (5.4)	4 (2.6)	17 (7.3)	0.034^a^
Aware	365 (94.6)	149 (97.4)	216 (92.7)	
**Sources of knowledge regarding premarital screening**				
Social media/Google	7 (1.8)	3 (2.0)	4 (1.7)	0.184
School/university	8 (2.1)	4 (2.6)	4 (1.7)	
Family/friends	104 (26.9)	47 (30.7)	57 (24.5)	
Health sectors/clinics	267 (69.2)	99 (64.7)	168 (72.1)	
**Opinion regarding best time for screening**				
During secondary school	48 (12.4)	15 (9.8)	33 (14.2)	0.634
At 18 years old	41 (10.6)	17 (11.1)	24 (10.3)	
Before engagement	255 (66.1)	103 (67.3)	152 (65.2)	
After engagement	42 (10.9)	18 (11.8)	24 (10.3)	

a Significant results (P value < 0.05).

**Table 3:** Relationships between screening test results and marriage decisions.

Test results	Overall = 386 n (%)	Did not marry = 153 n (%)	Did marry = 233 n (%)	*P*
Sickle cell trait (carrier)	325 (84.2)	121 (79.0)	204 (87.5)	0.057
β-thalassemia trait (carrier)	52 (13.5)	29 (19.0)	23 (9.9)	
Sickle cell disease	9 (2.3)	3 (2.0)	6 (2.6)	
β-thalassemia disease	0	0	0	

∗Significant results (P value < 0.05).

### Counseling

Approximately 23.1 % of the couples attended all clinical counseling sessions, whereas 76.9 % visited a counseling clinic at least once. The couples who attended all counseling sessions were more likely to decide not to marry than those who visited the counseling clinics only once (Table 4).

**Table 4:** Relationship between participants’ visits to counseling clinics and marriage decisions.

Variables	Overall = 386 n (%)	Did not marry = 153 n (%)	Did marry = 233 n (%)	*P*
**Attended all clinical counseling sessions**				
No	279 (76.9)	73 (47.7)	224 (96.1)	0.001^a^
Yes	89 (23.1)	80 (52.3)	9 (3.9)	
**Effect of counseling on marriage decision**				
No	176 (45.6)	49 (32.0)	127 (54.5)	0.001^a^
Yes	210 (54.4)	104 (68.0)	106 (45.5)	
**Attendees at counseling**				
One partner alone	201 (52.1)	123 (80.3)	78 (33.5)	0.001^a^
Both partners	100 (25.9)	14 (9.2)	86 (36.9)	
Partners and their families	85 (22.0)	16 (10.5)	69 (29.6)	
**Decision-maker regarding marriage**				
One partner alone	304 (78.8)	71 (46.4)	0	0.001^a^
Both partners (couple)	68 (17.6)	68 (44.4)	233 (100)	
Couple and their families	14 (3.6)	14 (9.2)	0	
**When the decision was made**				
Immediately after positive result	189 (49.0)	75 (49.0)	114 (48.9)	0.001^a^
After counseling session	124 (32.1)	65 (42.5)	59 (25.3)	
After discussion with partner/family	73 (18.9)	13 (8.5)	60 (25.8)	

a Significant results (P value < 0.05).

### Reasons for deciding to proceed or not proceed with “At-risk” marriages

At-risk couples were asked to identify their primary reasons for deciding to proceed or not proceed with marriage. They identified multiple reasons (Table 5, Table 6).

**Table 5:** Reasons why at-risk couples did not proceed with marriage despite knowing the risk of bearing affected offspring.

Reasons	n (%)
The importance of having healthy children	133 (86.9)
I Know the high hereditary risk of having affected children among couples with the traits	84 (54.9)
I Know couples who have children with SCD and thalassemia	89 (58.2)
My family convinced me to not proceed	39 (25.5)
Counseling sessions in the clinics convinced me to not proceed	69 (45.1)

**Table 6:** Reasons why at-risk couples proceeded with marriage despite knowing the risk of bearing affected offspring.

Reasons	n (%)
Belief that illness is determined by God's will	165 (70.8)
The relationship between the couple	135 (57.9)
Knowing couples with inherited blood diseases who have healthy children	107 (45.9)
Thinking that the chance of transmission of inherited disease was small	87 (37.3)
Planning to have in vitro fertilization	49 (21.0)
The counseling was not convincing	24 (10.3)
Financial marriage arrangements were completed before premarital testing	22 (9.4)
Fear of inability to marry a healthy partner	17 (7.3)
Knowing couples who have affected children and adapted to their disease	14 (6.0)
Pressure from family	13 (5.6)
Planning not to have children	8 (3.4)
Avoiding loneliness	5 (2.1)
Not realizing the seriousness of inherited blood disorders	1 (0.4)

## Discussion

The premarital screening program in KSA became mandatory in 2004. Despite the success of the program in reaching the target group, the percentage of married couples identified as being genetically incompatible in the current study was high (60.4 %). However, this outcome was expected, because couples may decide to marry after the testing. Therefore, the present study investigated determinants of at-risk couples’ decisions to proceed or not proceed with marriage. Marriage was found to be more prevalent among couples at risk of having a child with SCD than β-thalassemia. Similar results have been observed in the Turkish premarital screening program,^5^ thus suggesting that the medical complications of SCD are less severe than those of β-thalassemia.

This study further indicated that most genetically incompatible couples at risk of having children with SCD or β-thalassemia were from the eastern, southern, and western parts of Jazan. This geographic concentration is explained by the historical and cultural relationships among most couples in the Jazan region, including the contemporary degree of consanguinity in the province^6^ and the potential for hemoglobin mutations.^7^ However, consanguinity was not a significant predictor of at-risk marriage in this study.

In the past, the average age at marriage in KSA was approximately 22 years.^8^ In the present study, the average age of the male participants was 31, and that of the female participants was 26. Nevertheless, the couples’ ages and age differences between partners were unrelated to their marriage decisions. Most (64.2 %) of the participants had completed primary and secondary education, and 35.2 % had a university education. However, no significant association was found between education level and marriage decisions, as expected, because the Saudi population is generally educated.

The participants who were already married, divorced, or widowed, or who had children during the screening period, tended to decide to accept the risks associated with at-risk marriage. These findings are similar to those from a study conducted in Bahrain.^6^ Because SCD causes severe medical symptoms and complications, individual families and communities are often aware of the seriousness of this disease.^9^ The participants in this study who were aware of the risk of SCD and its effects on children's lives were more likely to decide not to marry than those who lacked such awareness. Additionally, the participants with a family history of SCD had a higher probability of not marrying than those without such history.

Most participants were aware of the importance of early premarital testing. The best time for such testing was believed to be before the couple's engagement by approximately 66.1 % of participants and during secondary school by 12.4 % of participants. Similar opinions have emerged in previous Saudi studies exploring public attitudes toward premarital testing and counseling.^10^^,^^11^ Because the premarital screening program is mandatory in KSA, the participants' main source of knowledge was healthcare facilities and clinics. However, to broaden the reach of such knowledge, educational efforts should also target television, radio, and social media.

Approximately 23.1 % of the couples attended all clinical counseling sessions, and 76.9 % visited a counseling clinic at least once. Couples who attended all counseling sessions were more likely to decide not to marry than those who visited a counseling clinic only once. This finding reflects the positive effects of counseling sessions on marriage decisions among at-risk couples. Most participating couples believed that illness is determined by God, and this factor was found to be the most significant influence on their decisions to marry. Religion appeared to enhance couples' acceptance of the possibility of their having a sick child. This finding might be attributable to the strong influence of Islamic rules and regulations on all aspects of Saudi people's lives.

Countries with high prevalence of inherited hemoglobinopathies, such as Cyprus and Iran, have implemented nationwide genetic screening and counseling programs that offer valuable models for decreasing at-risk marriages. Cyprus, adopting a model of mandatory screening with strong enforcement, has successfully decreased new cases of β-thalassemia to near zero through a mandatory premarital screening and genetic counseling program established in the 1970 s. Key elements of the Cypriot approach include free and mandatory testing for all couples before marriage; comprehensive public education campaigns that raised awareness across all social levels; and strong counseling and community pressure to prevent high-risk unions among couples found to be at-risk, although these couples are not legally forbidden to marry. This success reflects a national commitment, strong genetic literacy, and community-level support for prevention. Iran, adopting a religious and medical integration in genetic policy, implemented a national premarital screening program in the 1990s. This program particularly targeted β-thalassemia and led to a significant decline in affected births. Distinct features of the program include mandatory premarital testing for the thalassemia trait; free or subsidized access to screening and genetic counseling in public clinics; and the development of regional genetic centers to increase access, particularly in rural areas. Iran's experience highlights how religious alignment with medical goals can help normalize genetic interventions in conservative societies.

## Conclusion

In Jazan province, KSA, SCD is highly prevalent, and marriage among genetically incompatible individuals at risk of having children with either SCD or β-thalassemia is likewise highly prevalent (60.4 %). Despite the positive effects of the province's health services' counseling regarding this risk, many social, cultural, and religious factors influence at-risk couples' decisions to marry. Therefore, in vitro fertilization and pre-implantation genetic testing are recommended for at-risk couples in the province. Despite their importance, accessibility to these services remains limited by many barriers including high costs, as well as the need for specialized laboratories, trained embryologists, and genetic counselors, which are lacking in the Jazan region. Furthermore, these services are culturally unacceptable to many couples who are unaware of the utility of genetic testing and have misconceptions regarding their safety, success rates, and moral implications. A limitation of this study is that some factors were not statistically significant but might still have influenced marriage decisions. Another limitation is that the tables contain multiple variables, which may have affected the clarity and interpretation of the results.

## Ethical approval

The Jazan Health Ethics Committee (reference number: H-10-Z-073) granted ethical approval (No. 2449) for the study to be conducted, and the research complied with the Declaration of Helsinki.

## Consent

Informed consent was obtained from all participants before participation in this study.

## Authors contributions

All authors contributed equally to the conception and design, analysis of data, and approved the final version of the manuscript. All authors have critically reviewed and approved the final draft and are responsible for the content and similarity index of the manuscript.

## Data sharing statement

The data supporting the findings of this study are available on request from the corresponding author, Mohammed Badedi.

## Source of funding

This research did not receive any specific grant from funding agencies in the public, commercial, or not-for-profit sectors.

## Conflict of interest

The authors have no conflicts of interest to declare.
